# Life‐history traits of *Tubastraea coccinea*: Reproduction, development, and larval competence

**DOI:** 10.1002/ece3.6346

**Published:** 2020-06-19

**Authors:** Bruna L. P. Luz, Maikon Di Domenico, Alvaro E. Migotto, Marcelo V. Kitahara

**Affiliations:** ^1^ Coastal and Ocean Systems Graduate Program Federal University of Paraná Pontal do Paraná Brazil; ^2^ Center for Marine Biology University of São Paulo São Sebastião Brazil; ^3^ Institute of Marine Science Federal University of São Paulo Santos Brazil

**Keywords:** invasive species, life cycle, management, offspring, *Tubastraea*

## Abstract

The sun coral *Tubastraea coccinea* Lesson, 1829 (Dendrophylliidae) is a widely distributed shallow‐water scleractinian that has extended its range to non‐native habitats in recent decades. With its rapid spread, this coral is now one of the main invasive species in Brazil. Its high invasive capability is related to opportunistic characteristics, including several reproductive strategies that have allowed it to disperse rapidly and widely. To better understand the reproductive biology of *T. coccinea* and aid in developing management strategies for invaded areas, we investigated aspects of its reproductive performance and life cycle, including the effects of colony size, seawater temperature and salinity, and lunar periodicity on offspring production and larval metamorphosis competence. A total of 18,139 offspring were released in different developmental stages, mainly from the larger colonies, which also produced larvae with longer competence periods. The main reproductive peak occurred during the First Quarter and New Moon phases and was highest in water temperatures around 26°C. Together, these results help to explain the rapid expansion of *T. coccinea* into non‐native habitats such as the Caribbean and southwestern Atlantic, and will inform actions of the recent Brazilian National Plan for the prevention, eradication, control, and monitoring of sun corals.

## Graphical abstract

*Tubastraea coccinea* is a wide distributed shallow‐water scleractinian coral that has been rapidly increasing its range to non‐native habitats. Its invasive success is related to opportunistic characteristics. Herein, we investigate some aspects from its reproductive performance and life cycle, including the effect of biotic and abiotic factors on offspring production and larval metamorphose competence.

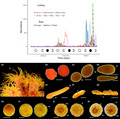

## 1 INTRODUCTION

The life history of a species, population, or individual refers to the timing and magnitude patterns of their major life events, such as maturation, reproduction, and longevity (Hughes & Leips, 2017). In general, life‐history traits are based mainly on quantitative and demographic properties, such as the number of offspring and size‐specific reproductive investment. Together, these traits are directly related to two primary components of fitness, survival and reproduction (Braendle, Heyland, & Flatt, 2011). Most life‐history theories attempt to explain how evolution modifies stage‐specific stages to maximize fitness, given the selection processes imposed by ecological challenges (Hughes & Leips, 2017; Stearns, 2000). Because organisms have limited resources and must allocate them to different functions (e.g., growth, reproduction, survival, and maintenance), trade‐offs and constraints that maximize reproductive success have been assessed in many studies (e.g., Braendle et al., 2011). By imposing specific opportunities for and constraints on reproduction, the biophysical properties of seawater and the connectivity of marine habitats are the main physical factors affecting marine organisms (Heyland, Degnan, & Reitzel, 2011). Because fitness is critical for the dispersal and evolution of sedentary organisms (Isaeva, Akhmadieva, Aleksandrova, Shukalyuk, & Chernyshev, 2011; Ritson‐Williams et al., 2009; Shikina & Chang, 2016; Whalan, Johnson, Harvey, & Battershill, 2005), these organisms have developed a diverse array of reproductive strategies and complex life histories (Braendle et al., 2011). One remarkable dispersal strategy is via larvae that may have direct or indirect development, the latter of which is involved in metamorphosis, a transformation from the larval to juvenile stage (Bishop, Huggett, Heyland, Hodin, & Brandhorst, 2006; McEdward, 2000).

In addition to the factors that influence the final stage of larval transport, such as suitable settlement sites and the mechanisms underlying metamorphosis (Pineda, Hare, & Sponaugle, 2011), larval dispersal involves spawning, transport, and survival. However, natural and human‐induced environmental disturbances are altering the historical patterns of reproduction, dispersal, and recruitment (Baker, Glynn, & Riegl, 2008; Crabbe, 2008; Glynn, Colley, Carpizo‐ituarte, & Richmond, 2017; Graham, Baird, Connolly, Sewell, & Willis, 2017; Nyström, Folke, & Moberg, 2000). In times of ever‐increasing abiotic challenges, knowledge of life‐history traits related to reproduction, larval dispersal, and genetic variation is especially important for scleractinian corals, which are the major builders of coral reefs (Sorek & Levy, 2014).

Similarly to many other cnidarians, scleractinian corals display a variety of asexual and sexual reproductive strategies (Fautin, 2002; Harrison, 2011; Richmond, 1997; Sherman, Ayre, & Miller, 2006; Ward, 1992). Asexual strategies including budding, fission, polyp bailout, and fragmentation followed by regeneration seem to be widespread (Cairns, 1988; Capel, Migotto, Zilberberg, & Kitahara, 2014; Highsmith, 1982; Luz et al., 2018; Sammarco, 1982). Sexual strategies of scleractinian corals involve either release of gametes into the water column (broadcasting) or releasing fully mature larvae as a result of self‐fertilization or outcrossing (brooding). Some brooder species release their offspring as fertilized eggs or embryos (Vermeij, Sampayo, Bröker, & Bak, 2004) or produce planulae asexually (Ayre & Resing, 1986; Sherman et al., 2006). Although shallow‐water zooxanthellate scleractinians are well known for their synchronized mass‐spawning events triggered by lunar and seasonal periods (Sorek & Levy, 2014), some species reproduce year‐round (e.g., *Tubastraea coccinea*; Glynn et al., 2008) or asynchronously for prolonged periods (e.g., *Turbinaria reniformis*; Harrison et al., 1984; Rapuano et al., 2017).

The morphological and molecular characteristics of the first developmental stages of both broadcasting and brooding scleractinians have been documented for several species (e.g., Fadlallah, 1983; Glynn et al., 2017; Hayward et al., 2011; Okubo, Hayward, Forêt, & Ball, 2016; Okubo et al., 2013; Strader, Aglyamova, & Matz, 2018). In general, newly emerged larvae have just completed gastrulation and are round, fragile, and motionless. Mature larvae are active and possess an elongated ciliated body with ectoderm, mesoglea, and endoderm surrounding a central coelenteron (Fadlallah, 1983). These planula larvae may disperse and recruit at long distances from or close to their parent colonies (Gleason & Hofmann, 2011). The capacity and scale of dispersal of scleractinian larvae are still unclear (Ayre & Hughes, 2000; Richmond, 1987). Overall, the transport of these larvae depends on multiple abiotic and biotic variables acting simultaneously, including currents (Wood et al., 2016), topography (Willis & Oliver, 1990), and the length of the pelagic larval period (Shanks, 2009). This last depends mainly on the period of larval competence, which is the ability to settle/attach and metamorphose into the primary polyp stage in response to environmental cues (Ben‐David‐Zaslow & Benayahu, 1998; Bishop et al., 2006; Gleason & Hofmann, 2011; Strader et al., 2018; Strathmann, 1986). These signals vary widely intra‐ and interspecifically, even across closely related species (Hodin, 2006). Besides, unless the period of larval competence is extremely short, dispersal is accompanied by growth and development, supported either by energy reserves from the mother colony, if azooxanthellate; or by nutrients from symbiotic photosynthetic dinoflagellates (Symbiodiniaceae), if zooxanthellate.

Competent larvae of scleractinian corals usually exhibit presettlement behaviors such as elongation, switching from swimming to crawling, and aboral substrate attachment (Fadlallah, 1983; Strader et al., 2018). Larvae may settle and then re‐enter the water column multiple times before they finally attach and undergo metamorphosis to the benthic life form (Eckman, 1996). In the absence of a suitable environment, nonfeeding larvae enter a state of low metabolism and may postpone metamorphosis without affecting their postsettlement fitness (Graham, Baird, & Connolly, 2008; Graham, Baird, Connolly, Sewell, & Willis, 2013; Graham & Nash, 2013). However, metamorphosis is an energy‐demanding process, especially due to the initiation of calcification and synthesis of new proteins, and the available energy for metamorphosis tends to decrease with planula age (Edmunds, Cumbo, & Fan, 2013; Richmond, 1987; Rodriguez, Sedano, García‐Martín, Pérez‐Camacho, & Sánchez, 1990; Sewell, 2005; Strader et al., 2018; Wendt, 2000). Therefore, azooxanthellate coral larvae such as those of *T. coccinea*, which do not receive nutrition from symbiont photosynthetic algae, may deteriorate and perish if this process is not accomplished within their competence period. Alternatively, although their survival and reproduction capabilities are still unclear, a few scleractinian species can undergo metamorphosis before settlement, extending their planktonic life by feeding in the water column (Mizrahi, Navarrete, & Flores, 2014; Richmond, 1987).

*Tubastraea coccinea* and some of its congeners (Dendrophylliidae, Scleractinia) invaded the Atlantic Ocean in the 1940s as biofouling on ships and oil and gas platforms (Cairns, 1994; Creed et al., 2017). *Tubastraea coccinea*, popularly known as the sun coral, has established populations in the Caribbean, Gulf of Mexico, and over 3,500 km of the Brazilian coast (Boschma, 1953; Cairns, 2001; Castro & Pires, 2001; Costa et al., 2014; Fenner, 1999, 2001; Fenner & Banks, 2004; de Paula & Creed, 2004; Romano & Cairns, 2000; Sammarco, Porter, & Cairns, 2010; Vaughan & Wells, 1943). Several of these invaded areas are experiencing economic and environmental impacts from this coral (Creed et al., 2017; Luz & Kitahara, 2017). One of the important reasons for the invasiveness and rapid spread of *T. coccinea* along the southwestern Atlantic coastline is suggested to be the occurrence of multiple primary (Capel et al., 2017) and secondary (Capel, Creed, Kitahara, Chen, & Zilberberg, 2019) invasions, along with its diverse reproductive strategies, early maturity (Fenner & Banks, 2004; Glynn et al., 2008), rapid growth, and high recruitment rate (Costa et al., 2014; Lages, Fleury, Menegola, & Creed, 2011). The remarkable regenerative capacity of this species appears to be another factor in its invasion success (Luz et al., 2018).

Information regarding *T. coccinea* life‐history traits that potentially maximize its fitness in non‐native habitats, such as abiotic and biotic processes that affect larval dispersal, is still lacking. Therefore, we investigated the effects of colony size, temperature, salinity, and lunar periodicity on the reproductive performance of *T. coccinea* during its main annual reproductive event. We also evaluated the succession of developmental stages and larval competence of *T. coccinea*.

## 2 MATERIALS AND METHODS

### 2.1 Sampling and specimen maintenance

Ten colonies of *T. coccinea* were collected by snorkeling, at the Ilhabela Yacht Club, São Paulo State, Brazil (23°46′20″S, 45°21′20″W), in December 2016, and kept in separate 2‐L open‐water system aquaria under environmental temperature at the Centre for Marine Biology (CEBIMar), University of São Paulo. Embryonic stages and larvae released from each colony were sampled once a day and placed in separate aquaria according to their respective release dates. These larvae were monitored every 24 hr for sampling different ontogenetic stages (newly settled, settled, early metamorphosis, metamorphosed, and recruit), which were then transferred to new aquaria (300‐500 ml) according to the stage (also kept separated by parent colony). The time for larvae to reach each developmental stage was tracked.

Parent colonies were fed every other day with 50 ml of freshly collected zooplankton ranging from 50 to 200 µm in diameter. All other ontogenetic stages were kept without food in closed water systems filled with 20 µm‐filtered seawater at 24°C, which was changed every 72 hr.

### 2.2 Offspring production as a function of biotic and abiotic traits

To assess the reproductive performance of *T. coccinea* and the potential effects of biotic and abiotic factors on its fitness, offspring produced from 10 colonies were monitored for 91 days (15 December–15 March), which coincides with its main reproductive period in the southwestern Atlantic (de Paula, Pires, & Creed, 2014) and also in the eastern Pacific (Glynn et al., 2008). During the experiment, we tested the number of offspring released (number of embryos and larvae per day) as the response variable, and lunar periodicity, temperature, and salinity as explanatory variables. Temperature and salinity were measured with a YSI Model 30 Handheld Conductivity, Salinity, & Temperature meter, once a day in surface water at the location where the water used in the experiment was obtained.

Our response variable showed an asymmetrical distribution and heteroscedasticity, tested with the Bartlett test (Bartlett, 1937) and Shapiro–Wilk normality test (Royston, 2006). We therefore performed nonparametric analyses with the Kruskal–Wallis test (Conover, 1980) and the Spearman rank correlation coefficient. We used Dunn's Kruskal–Wallis multiple comparisons (Dunn, 1964) to assess significant sources of variation related to lunar periodicity. Although *T. coccinea* has early maturity (Fenner & Banks, 2004; Glynn et al., 2008), larger colonies may show better reproductive performance (Stearns, 1992). To test this hypothesis, a Spearman rank correlation coefficient and linear regression analyses were performed to determine whether the reproductive potential (number of offspring released) was correlated to colony size. Colony size was measured by the volume and number of polyps. As the colony of *T. coccinea* is phaceloid, with an overall convex shape, its volume was calculated using the truncated pyramid formula:

$$v=\frac{h}{3}\times \left(A+\sqrt{A\times a}+a\right)$$

where *v* = volume; *h* = height; *A* = base side; and *a* = top side.

As the colony volume and number of polyps were correlated (*r*s = .68; *N* = 10; *p* = .035), the “size” effect on offspring production was measured only by polyp number, as this characteristic is easier to estimate in the field for management purposes. Last, to check whether the life history can determine reproductive performance, colonies were sorted by size (in quartiles; small < 38 polyps, large > 52 polyps, and medium = 38–52 polyps) and the variation in larval release was measured by using the nonparametric Kruskal–Wallis test (Conover, 1980). All statistical analyses were performed in R v.3.2. The Agricolae package with “BH” as the adjustment method was used for the Kruskal–Wallis analyses (Benjamini & Hochberg, 1995).

### 2.3 Life cycle: from larva to recruit

Offspring development from 10 colonies of *T. coccinea* was examined and the offspring morphology and behavior described, including those larvae that underwent metamorphosis in the water column. For this, all newly released larvae were sampled from each colony during different planulation events and monitored daily. Images of the developmental stage were taken with a Sony Handycam HDR‐XR520, coupled to a Zeiss Stemi 2000‐C stereomicroscope. Development was tracked to acquire data on the duration of the different ontogenetic stages and also to estimate larval longevity and competence.

As ontogenetic development and larval competence are individual traits (Eckman, 1996), which in turn may be a response of phenotypic plasticity and different amounts of energy provided to the offspring (Zera & Harshman, 2011), we examined the intraspecific variation in the time required to pass through each developmental stage under laboratory conditions (24°C). For this purpose, the development and larval competence were evaluated according to larval age and life history (sorted by colony size), using the Spearman rank correlation coefficient and nonparametric Kruskal–Wallis test (Conover, 1980), respectively. All raw data used in the present study can be found at: https://doi.org/10.5061/dryad.zw3r2285p

## 3 RESULTS

### 3.1 Environmental conditions

The seawater temperature ranged from 24 to 30°C and the salinity from 33 to 35. The lowest mean temperature and salinity were recorded in December (24.65 ± 0.4°C and 33.64 ± 0.64, respectively). February had the highest temperature (26.47 ± 1.17°C) and intermediate salinity (33.94 ± 0.42). January and March had intermediate temperatures (January: 26.05 ± 1.31; March: 26 ± 0.61) but the highest salinities (January: 34.1 ± 0.45; March 34.43 ± 0.64).

### 3.2 Reproductive performance

**FIGURE 1:**
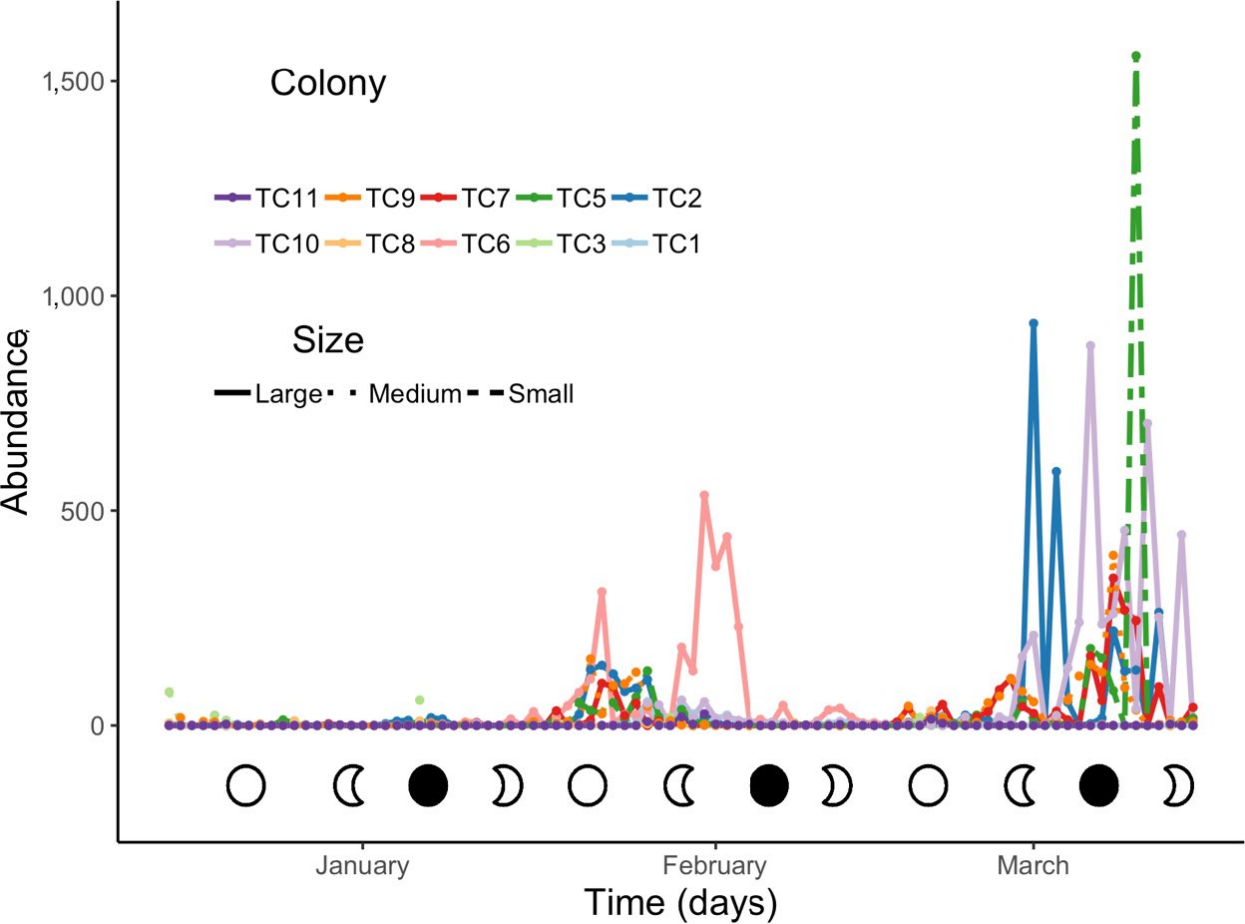
*Tubastraea coccinea* offspring abundance in relation to the number of colonies (*n* = 10) and colony size (*n* large = 4; *n* medium and small = 3 each) between 15 December 2016 and 15 March 2017

**FIGURE 2:**
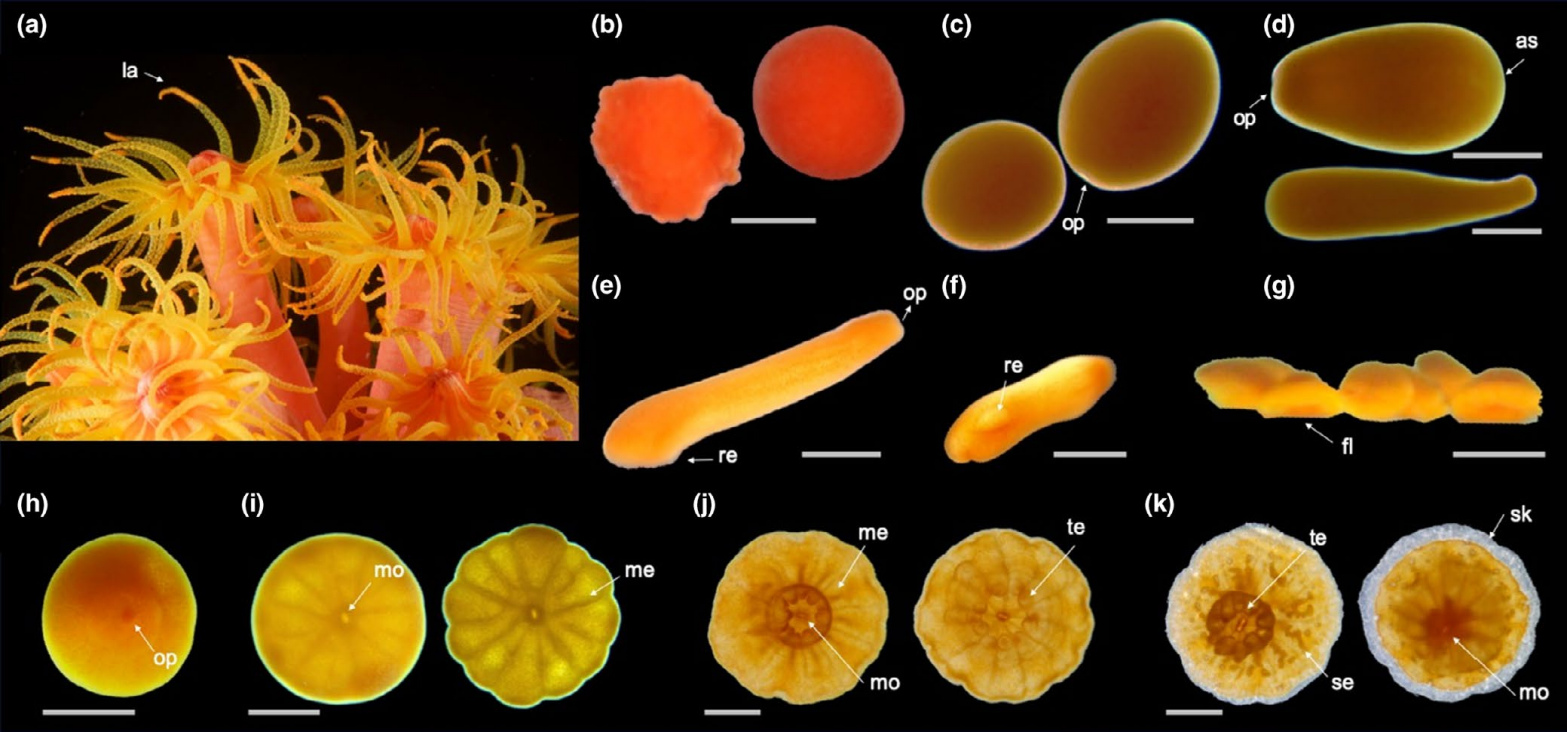
Developmental stages of *Tubastraea coccinea*: from adult to recruit. Morphological aspects of (a) adult colony with larvae in the tentacles; (b) embryos; (c) newly formed larvae; (d) mature larvae, contracted and elongated; (e) newly settled larva; (f) larva detached from substrate; (g) lateral view of group of settled larvae; (h) settled larva; (i) near metamorphosis larva; (j) metamorphosed larva; and (k) recruit (h–k show oral sides). Arrows indicate the following: la—larvae; op—oral pore; as—aboral side; re—reforming to flattened form (fl); mo—mouth; me—mesentery; te—tentacle; se—calcareous septa; and sk—skeleton. Scale bars represent 0.5 mm

Reproductive activity was observed over the 3 months of the experiment. Two main peaks of embryo and/or planula release were recorded (Figure 1): a smaller peak at the end of January and early February, and a larger peak in early March. Ten colonies of *T. coccinea* released a total of 18,139 offspring (Figure 2a), including 442 embryonic stages (Figure 2b) and 17,697 larvae (newly released and mature larvae, Figure 2c,d, respectively). The maximum number of larvae released during a single event (24 hr) was 1,561 and was from a small colony (17.14 cm^3^; 34 polyps).

Planulation events occurred during different periods of the day but preferentially at night, with different stages of larval development (newly formed and mature larvae) frequently being spawned together by the same colony. Aggregations of newly formed and mature larvae were observed in the tentacles of the mother colony (Figure 2a), from where some of them were released through a small pore at the tip of the tentacle (Online Resources 1 and 2). In some cases, mature larvae were observed swimming freely inside the mother colony (Online Resource 3) and more sporadically passing actively into and out of the mouth of the mother polyp. For those offspring not “expelled” from the mother colony, neither through tentacles nor from a “water jet” (Online Resources 4, 5, and 6—see also Online Resource 7 for larvae actively swimming out of the mother colony), active movement of the mother polyp's mesenteries exposed the planulae to the water column (Online Resource 8).

**FIGURE 3:**
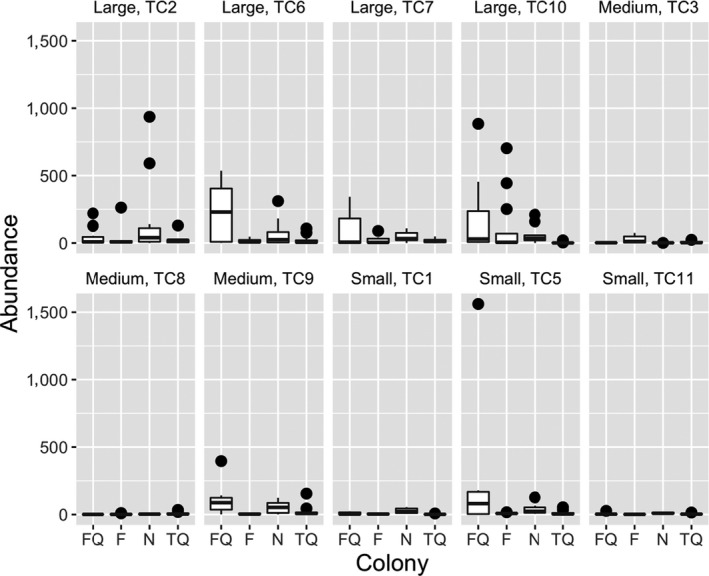
Number of offspring in relation to colony and lunar period. F, Full; FQ, First Quarter; *N*, New; TC, colony number; TQ, Third Quarter Moon phases

Larval release varied among lunar phases (Table 1; Figure 3), considering each phase beginning with the first day of each lunar phase. The highest numbers of larvae were released in the First Quarter (49%) and New Moon phases (31%), followed by the Full Moon phase (13%). Embryos were released mainly during the Third Quarter phase (67%), although their number did not differ significantly from other lunar periods. Although temperature and salinity were not measured every day and did not show a significant effect on planulation, higher numbers of larvae were released when the water temperature was 26°C, regardless of the salinity (79%; *N* = 10,456 larvae). In contrast, the number of embryos was correlated only with temperature (*r*s = .32; *N* = 50; *p* = .002); embryos were released more frequently in conditions of higher temperature and salinity.

**TABLE 1:** Kruskal–Wallis test and Dunn's post‐test for comparisons of the number of embryo cells and larvae released by *Tubastraea coccinea* with lunar periodicity and colony size (as number of polyps: small < 38; medium 38–52; large > 52)

Factor	Variable	KW	Dunn
Lunar periodicity			
	Embryos	Chi‐squared = 04.449, *df* = 3, *p* = .216	—
	Larvae	Chi‐squared = 39.149, *df* = 3, *p* < .001	N = FQ > F = TQ
Colony size			
	Embryos	Chi‐squared = 11.702, *df* = 8, *p* = .165	—
	Larvae	Chi‐squared = 17.023, *df* = 2, *p* < .001	L > M = S

Abbreviations: F, Full; FQ, First Quarter; L, large; M, medium; N, New; S, small; TQ, Third Quarter.

**FIGURE 4:**
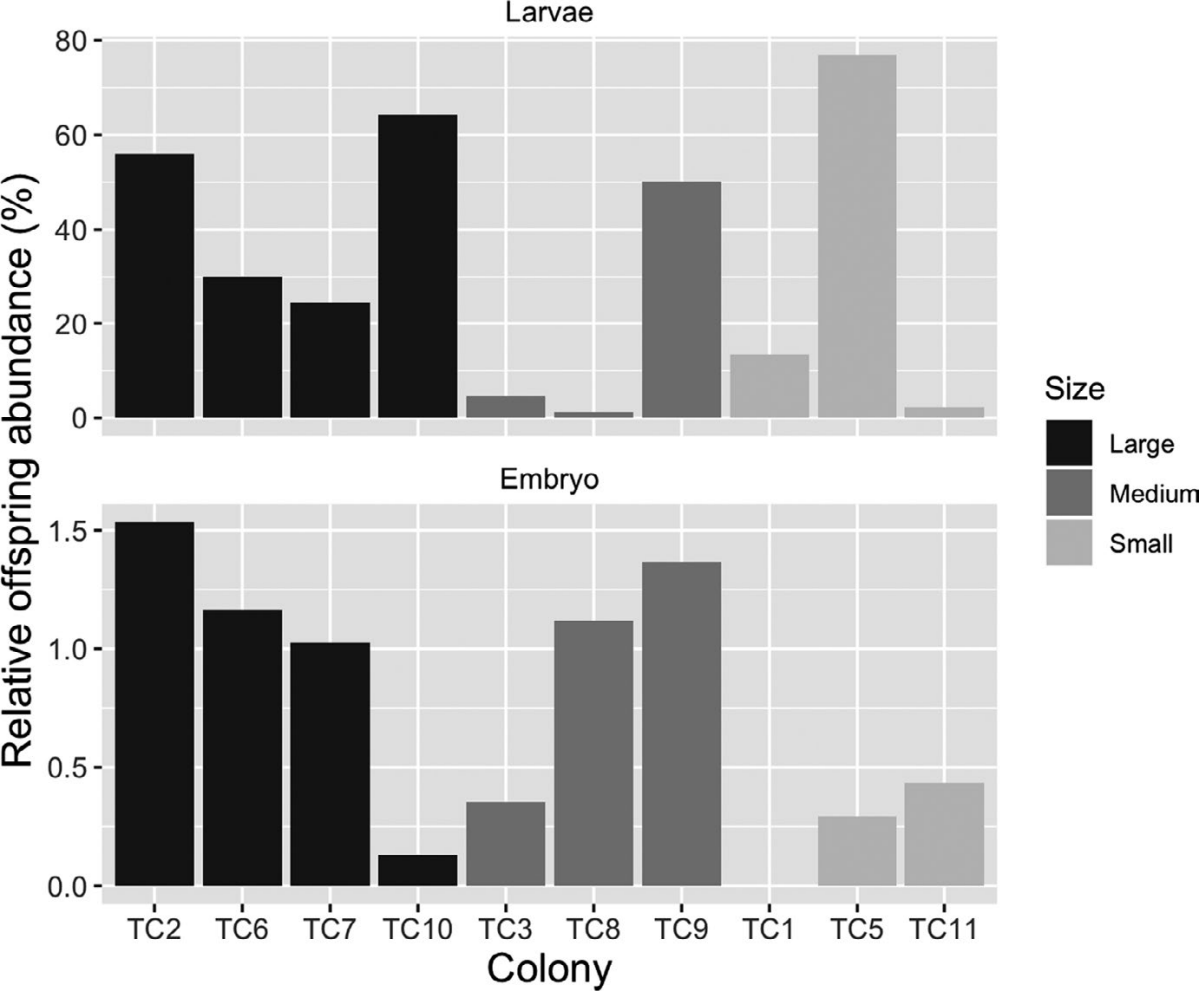
Number of offspring (larvae and embryos) per polyp, grouped by colony size: large (TC2, 6, 7, 10), medium (TC3, 8, 9), and small (TC1, 5, 11)

Regarding the effect of colony size on reproductive performance, the number of polyps was not significantly related to the number of larvae. Colonies of *T. coccinea* displayed a continuous and subtle trend toward asynchrony within peaks of high larval release (Figures 1 and 3). Furthermore, a significant variation in these events was observed when colonies were sorted by size, as a proxy of life history (Table 1; Figure 4). Overall, larger colonies (>52 polyps) produced more larvae (~70%) than medium (~14%) and small (~16%) colonies. On the other hand, the number of embryos released was correlated with the number of polyps (*r* = .68; *N* = 10; *p* = .035).

### 3.3 Life cycle and larval competence

*Tubastraea coccinea* breeds continuously throughout the year*,* but releases larvae mostly during its main reproductive period (Glynn et al., 2008; de Paula et al., 2014). Surprisingly, we observed colonies releasing offspring in different developmental stages (from embryos to mature larvae) simultaneously or within the same reproductive cycle. The released embryos (Figure 2b) were in several stages of embryogenesis, including morulas and spherical embryos with a closing blastopore. Although later embryos were able to resume development in the water column, in aquaria they could become trapped by the water surface tension and burst before reaching the larval stage. Released near formed larvae (Figure 2c) were motionless (Online Resource 9), round, and redder than mature larvae (Figure 2d), which ranged from yellowish orange to orange. Mature larvae were active, with high swimming capacity (using cilia and spinning around the oral–aboral axis), rapid body contraction/elongation, and eventually switching from swimming to crawling behavior and vice versa.

**FIGURE 5:**
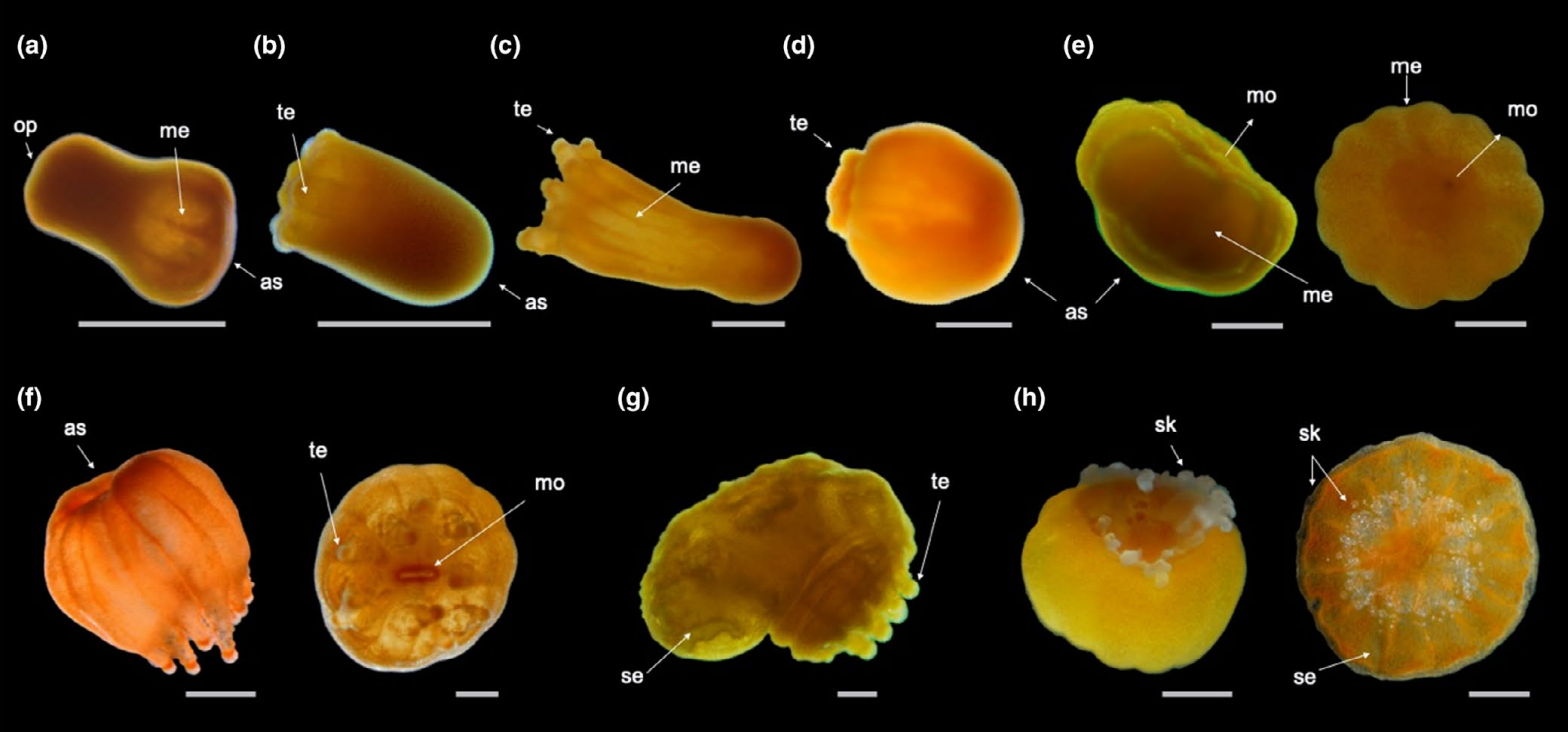
Developmental stages of *Tubastraea coccinea* in the water column: from larvae undergoing metamorphosis, before settlement. Morphological aspects of larva undergoing metamorphosis before settlement, in half of the body, either the (a) aboral or (b–d) oral side; (e) near metamorphosis; (f) metamorphosed; (g–h) recruits. Arrows indicate the following: op—oral pore; as—aboral side; mo—mouth; me—mesentery; te—tentacle; se—calcareous septa; and sk—skeleton. Scale bars represent 0.5 mm

Newly settled larvae (Figure 2e) were those that attached to the substrate and began to undergo metamorphosis. These larvae had a deformation on their aboral side, which was in contact with the substrate. This deformation was retained by the larvae that settled but later returned to the water column (Figure 2f), and in such cases, they often underwent metamorphosis before reattaching to the substrate (Figure 5).

Following attachment, which marked the end of the motile stage and the beginning of benthic life, the settled individual promptly lost the spherical/pear shape and acquired a triangular form. The latter was characterized by a flattened aboral side (Figure 2g) and an oral pore at the other end (Figure 2h), which later originated the mouth. After it settled, the near metamorphosed polyp was radially symmetrical and displayed a centrally located mouth encircled by mesenteries, which were visible through the transparent body wall (Figure 2i). The appearance of tentacles and skeleton marked the metamorphosed (Figure 2j) and recruitment (Figure 2k) stages, respectively. During metamorphosis, tentacles emerged as small balls and then elongated, achieving full development mainly at the recruitment stage, when batteries of nematocysts were formed. Although skeleton secretion began to be visible during the recruitment stage, the synthesis of the extracellular organic matrix preceded this stage, once a thin tissue surrounding the polyp, where skeletal crystals were later deposited, was observed in the preceding ontogenetic stage.

**FIGURE 6:**
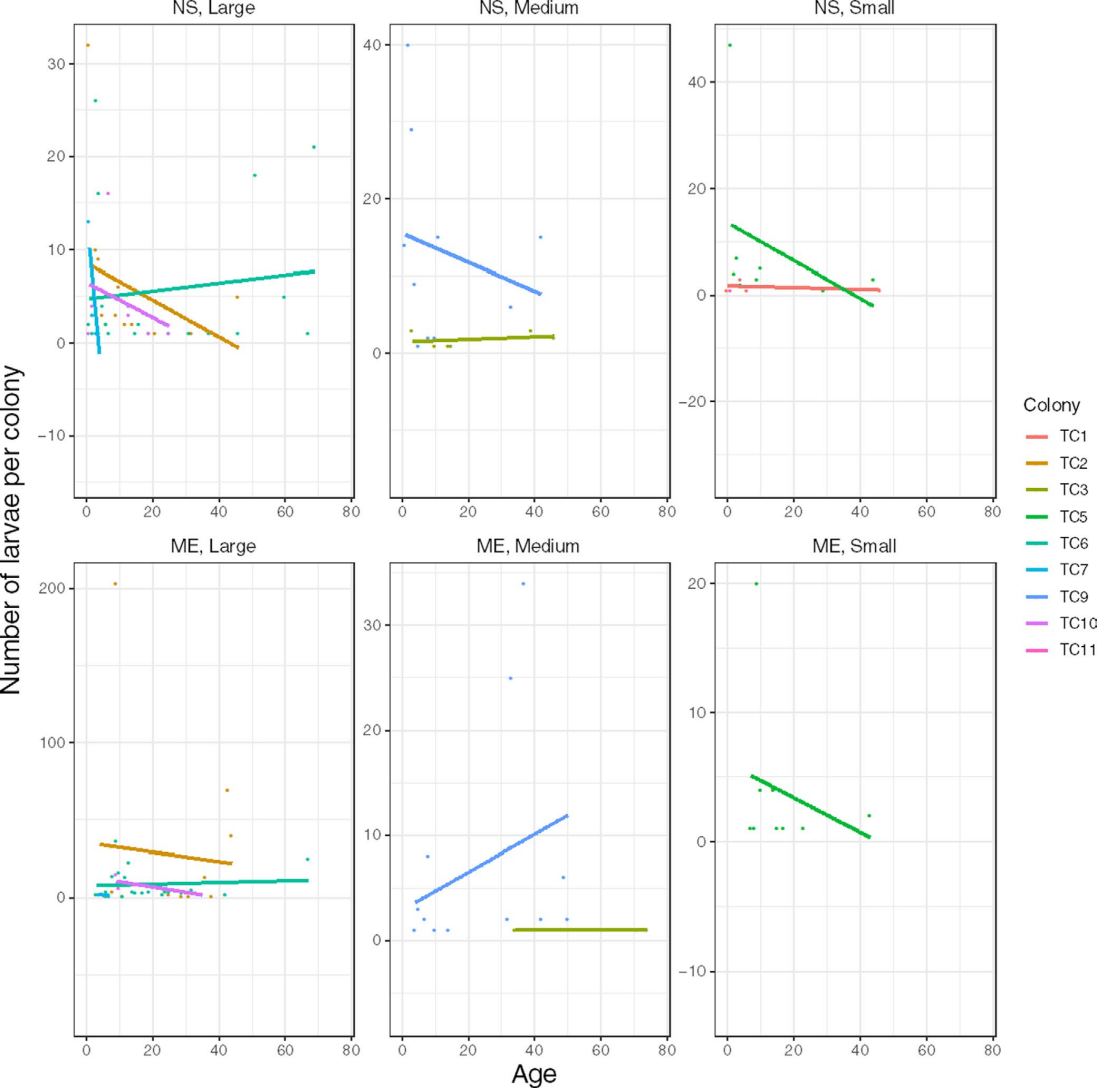
Relationship of *Tubastraea coccinea* larval competence and metamorphosis with size of mother colony (small, medium, and large) measured in relation to period of time (larval age) required to reach early developmental stages: NS—newly settled larvae; and ME—metamorphosed. Bands indicate 95% confidence interval

**FIGURE 7:**
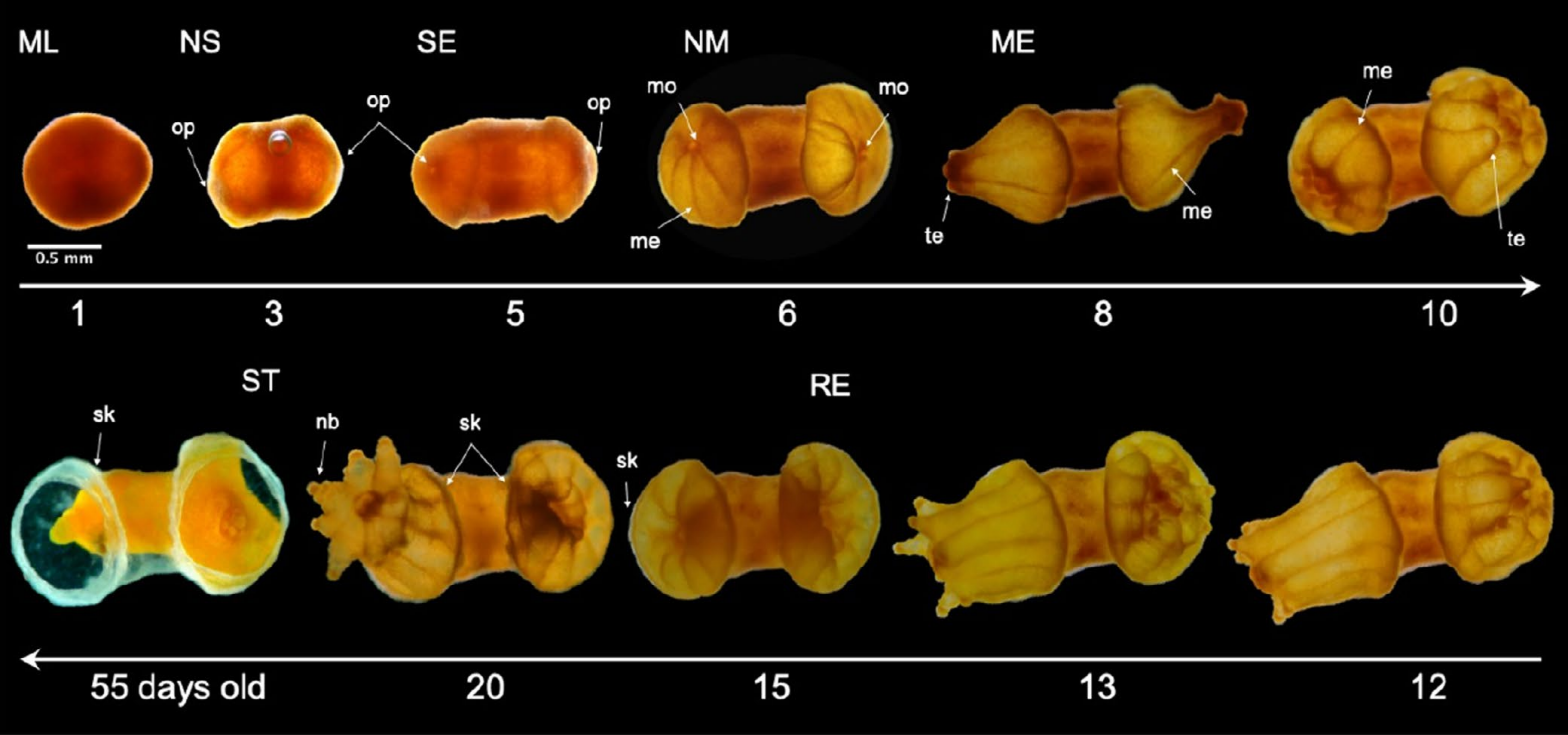
Colony of *Tubastraea coccinea* originating directly from a single larva, through the period of development. Morphological aspects of mature larva (ML), newly settled (NS), and settled with two oral pores (SE); near metamorphosis (NM) and metamorphosed (ME), with two early polyps; recruit (RE) secreting skeleton on its basal plate and around the calyx margin from each polyp emerged; and primary polyp retreated after environmental stress (ST), showing the skeleton growth pattern. Arrows indicate the following: op—oral pore; mo—mouth; me—mesentery; te—tentacle; nb—batteries of nematocysts; and sk—skeleton

The time required for mature larvae to reach the recruit stage under laboratory conditions differed among individuals (*p* = .9). Recruits were observed from the 8th day but were most abundant on the 33rd day (~37%; *N* = 203) after release. Larvae showed a varied competence period (Figure 6), with some starting to settle on the same day that they were released, although more often on the 2nd day (NS: ~23%; *N* = 477). The settled stage was reached mainly on the 3rd day (SE: ~20%; *N* = 408). Near metamorphosed and metamorphosed stages were observed mainly at the 4th (NM: ~27%; *N* = 371) and 10th days (ME: ~41%; *N* = 666) after larvae were released, respectively (Figure 7). However, several larvae were able to undergo metamorphosis even after 40 days (Figure 6); the longest competence period observed was 69 days. Some larvae survived in the water column through the entire period of the experiment (91 days). Therefore, *T. coccinea* larvae may remain in this state longer than 91 days, since the remaining larvae were healthy at the end of the experiment.

**FIGURE 8:**
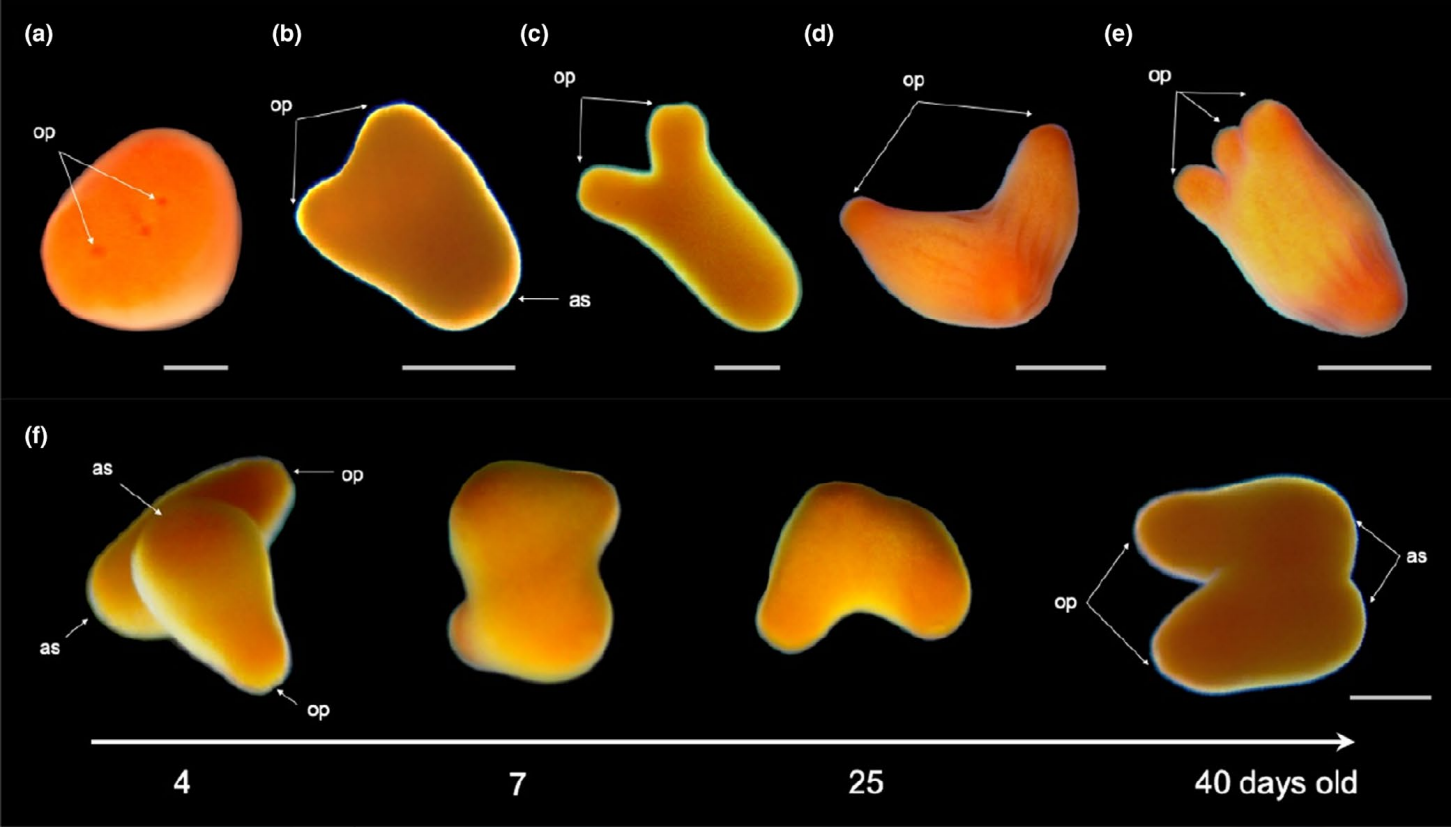
Alternative larval development of *Tubastraea coccinea* that may originate a small primary colony rather than a single primary polyp. Morphological aspects of near formed larvae with two or more oral pores (a), which developed into a mature larva with a “boomerang” body form (b–d) or with three distinct elongate “arms” (e); and fusion of two larvae over time (f). Scale bars represent 0.5 mm

**FIGURE 9:**
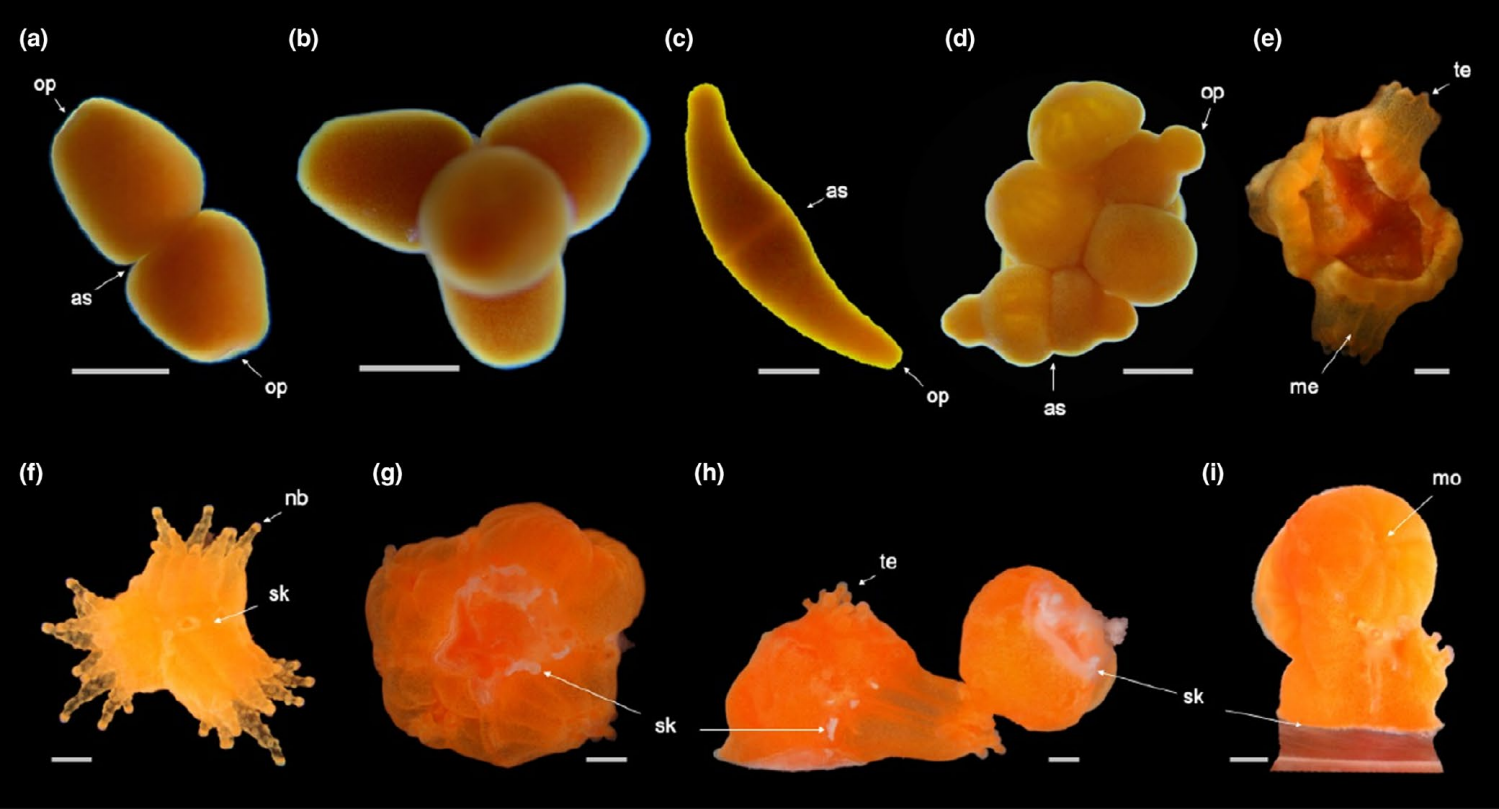
Chimeric larvae and colonies formed by one or more larvae that settled on another larva (a–c) or on an individual in a different stage of development (d), which are able to undergo metamorphosis (d, e) and secrete a skeleton on its basal plate (f–i) in the water column. Most of these recruits were able to attach to the substrate a second time (h, i). Arrows indicate the following: op—oral pore; as—aboral side; mo—mouth; me—mesentery; te—tentacle; nb—batteries of nematocysts; and sk—skeleton. Scale bars represent 0.5 mm

The larvae showed alternative life cycles and developmental stages: (a) larvae that underwent metamorphosis before settlement and had half of the body in the larval form, with the other half, usually the oral side, containing well‐formed mesenteries, a mouth, and early or well‐developed tentacles (Figure 5a–d); (b) near metamorphosis, metamorphosed, and recruits developed in the water column (Figure 5e,f, and g,h, respectively); iii) near‐larvae with two or more oral pores (Figure 8a), which developed into a mature, boomerang‐shaped larva (Figure 8b–d) or with three distinct elongate “arms” (Figure 8e); (d) fusion of two or more larvae (Figure 8f); (e) one larva that originated a small primary colony rather than a single primary polyp (Figure 7); and (f) one or more larvae that settled on another larva or individual in a different stage of development, forming a chimeric colony (Figure 9). Most of these water‐column recruits were able to attach to the substrate a second time and form a primary founder polyp or even a small colony.

## 4 DISCUSSION

### 4.1 Reproductive performance

Nowadays, as a result of its invasiveness capabilities, *T. coccinea* is the most widespread shallow‐water scleractinian coral species. It possesses several reproductive strategies that promote its dispersal, high abundance, and persistence in non‐native habitats, such as in the southwestern Atlantic, Gulf of Mexico, and Caribbean (Creed et al., 2017; Glynn et al., 2008). However, although the reproductive ecology of *T. coccinea* from native (Glynn et al., 2008) and non‐native habitats (de Paula et al., 2014) has been studied, there is a lack of information regarding planulation events and the influence of biotic and abiotic factors on this process. Here, we investigated the effects of colony size, temperature, salinity, and lunar periodicity on the reproductive performance of *T. coccinea*.

Overall, the larval production of the Brazilian invasive *T. coccinea* during its annual main reproductive period (18,139, of which 442 were embryonic stages and 17,697 larvae) was higher than the production estimated for colonies in the Galápagos (1,139.00 ± 31.33) and Panama (247.62 ± 3.78) (Glynn et al., 2008). Considering the alarming densities of *T. coccinea* in non‐native habitats (de Paula et al., 2014; Silva et al., 2014), which may increase more than 70% per year (Lages et al., 2011), there is no doubt that the asexual production of a large number of larvae (Capel et al., 2017, 2019) is aiding its successful spread and colonization of new habitats. These high densities lead to substantial changes in the structure and function of the native benthic community (de Paula et al., 2014; Silva et al., 2014; Silva et al., 2019). For example, at Búzios Island, *T. coccinea* and *Tubastraea tagusensis* cover the hard substrate at many locations, outcompeting native and endemic species such as *Palythoa caribaeorum* (Luz & Kitahara, 2017) and *Mussismilia hispida* (Creed, 2006), and also changing the soft‐bottom seascape (Capel, Creed, & Kitahara, 2020).

*Tubastraea coccinea* releases offspring at different times of the day, but preferentially at night, as also observed by Glynn et al. (2008). Although larvae are commonly released from the polyp's mouth, it was not uncommon to find large aggregations in the gastrovascular cavity, between the mesenteries near the oral disk, and inside tentacles, from the tip of which larvae could also be released (Figure 2a; Online Resources 1 and 2). While this is the first report of such behavior in *T. coccinea*, larva release via tentacle tips is not an exclusive feature of this species (Fadlallah, 1983; Harrison, 2011). For example, the brooder coral *Eusmilia fastigiata* may spawn gametes or release early‐stage embryos through a distal pore of its tentacle (Bastidas et al., 2005; Graaf, Geertjes, & Videler, 1999; Steiner, 1995); and *Stephanocoenia intersepta* shows intratentacular fertilization, that is, it keeps its eggs inside the tentacles to increase their exposure to spawned sperm and enhance fertilization success (Vermeij, Barott, Johnson, & Marhaver, 2010). In the sea pen *Umbellula lindahli*, mature eggs can be squeezed out through small tentacular pores as well (Tyler, Bronsdon, Young, & Rice, 1995).

We also found that *T. coccinea* releases offspring at different developmental stages (later embryos, and newly formed and mature larvae; Figure 1b,c,d, respectively). This behavior may be a result of overlapping gametogenic cycles, which are common in polyps of brooding soft and scleractinian corals such as *E. fastigiata* and *Anthelia glauca* (de Graaf et al., 1999; Kruger, Schleyer, & Benayahu, 1998). Although released embryos can continue their development in the water column, most of them perish, as also observed for *E. fastigiata* (de Graaf et al., 1999). This high mortality may be explained by the lack of cilia in these early embryos (not natant), which have a poorly defined cellular layer covering an indistinct yolky mass (de Paula et al., 2014). In aquaria, some of these fragile embryos float to the surface and are crushed by the force of the water surface tension.

### 4.2 Biotic and abiotic effects on offspring production

The reproductive pattern of *T. coccinea*, with a smaller peak of planulation during the end of January and early February, and a larger peak in early March, is consistent with that observed for eastern Pacific native populations (Glynn et al., 2008), and also for invasive populations at Rio de Janeiro, Brazil (de Paula et al., 2014). These events were correlated with lunar cycles (Figure 1), with higher larval abundance in the First Quarter and New Moon phases, while embryos were mainly released during the Third Quarter Moon phase. Although in smaller numbers, larvae were also released after the Full Moon. This synchronicity with lunar phases is similar to observations in Taiwan (Lin, 2005), Costa Rica, Panama, and the Galápagos (Glynn et al., 2008), and also for other brooding corals such as *Seriatopora hystrix* and *Pocillopora damicornis* (Fan, Li, Ie, & Fang, 2002).

Despite the influence of the lunar cycle, some intraspecific variations in the frequency and abundance of offspring release were observed (Figure 3). Colonies of *T. coccinea* have a continuous and subtle trend toward asynchrony within the peaks of higher offspring release. Most colonies (all small, three medium, two large) released more offspring over 17 days within one of the active reproductive periods. The exceptions were the medium (TC9) and large (TC2, TC7) colonies that had more than one peak, although with smaller numbers during the first peak (Figure 1).

The mean time between planulation peaks was around 12 days, which is shorter than the 6 weeks expected to release brooded larvae from new fertilizations (Glynn et al., 2008). This indicates that *T. coccinea* populations in invaded areas reproduce continuously, with overlapping different developmental stages of oocytes, spermatic cysts, and larvae, as previously observed for native and invasive colonies in the eastern Pacific and southeastern Brazil, respectively (Glynn et al., 2008; de Paula et al., 2014). On the other hand, the observation of newly formed and mature larvae being released simultaneously from the same colony, as well as the presence of larvae in the tentacles, suggest that *T. coccinea* may also be able to postpone releasing larvae until environmental conditions occur that maximize survival of its offspring.

Brooder corals typically have multiple planulation cycles per year, which may vary in timing among populations from different localities in response to environmental factors (Crowder, Lo, Weis, & Fan, 2014; Fan et al., 2002; Harrison & Wallace, 1990). *Tubastraea coccinea* is known to reproduce year‐round, with larvae being released mostly during warmer months in localities with well‐defined seasons (Glynn et al., 2008; de Paula et al., 2014). Although we did not measure the seawater temperature and salinity daily, the highest numbers of larvae were released at a seawater temperature of 26°C regardless of the salinity, while embryo cells were released mainly in higher water temperatures and salinity around 35.

Early gamete maturation and planulation events were previously observed for corals (e.g., *P. damicornis*) in periods of higher seawater temperatures, even over a single reproductive cycle (Crowder et al., 2014). Such a shift in timing can reduce larval survival, as in the zooxanthellate coral *Fungia scutaria* (Schnitzler, Hollingsworth, Krupp, & Weis, 2012). Therefore, our results indicate that the combination of higher temperatures with high salinity may not be suitable for *T. coccinea* larval development, or even induce premature spawning (i.e., the release of embryonic stages before their complete formation; reviewed by Loya & Rinkevich, 1980). On the other hand, sun corals may release embryos that after a few days develop into larvae in the water column (as seen in aquaria) as a possible reproductive strategy for increasing larval dispersal, as seen in several broadcaster species as a significant evolutionary trait that provides a balance against local mortality (Ritson‐Williams et al., 2009). Colony size is another factor that might trigger this reproductive effort, and then be determinant for intraspecific variation. Even colonies as small as two polyps are capable of producing eggs and larvae in similar proportions to colonies with up to 10 polyps; the relationship between planula number and colony size varies in different localities (Glynn et al., 2008). Here, we found no statistically significant trend between the number of polyps and the number of larvae. As all the colonies studied here were collected from the same location and are expected to be clones (Capel et al., 2017), the intraspecific variation in reproductive performance may be related to each colony's life history (e.g., previous stress events).

Overall, larger colonies (>52 polyps) showed better reproductive performance (Figure 4) than medium and small ones. An exception was a small colony (TC5) that had an exceptional spawning event during its second main reproductive peak (Figure 3). This event also influenced the estimation of the rate of offspring per range of colony size, which supports the hypothesis that intraspecific variance in offspring production can be determined by life history rather than by colony size. Since we measured the effect of only a limited range of colony sizes (20–91 polyps) on offspring release, further experiments are needed to confirm this hypothesis.

### 4.3 Life cycle and larval competence

The life cycle of corals includes a planktonic larval phase that is critical for the maintenance of adult populations (Gleason & Hofmann, 2011), by replenishing the local area with new genotypes or by spreading them over longer distances, supporting reef connectivity and enhancing genetic diversity (Ritson‐Williams et al., 2009). The combination of hydrodynamics and the time spent in the water column is the main mechanism that naturally drives the transport and dispersal of coral larvae (Shanks, 2009; Wood et al., 2016). Therefore, larval longevity (more than 91 days) and the extended period of competence (69 days), together with the high numbers of offspring of *T. coccinea,* confer a high dispersal ability on this invasive coral, which contributes to its rapid distributional expansion in invaded habitats.

Some of the *T. coccinea* larvae deviated from the idealized cycle expected for corals (Eckman, 1996; Harrison, 2011; Ritson‐Williams et al., 2009), which comprises a motile larval phase followed by a benthic phase of the recruit to adult stages (Figure 2). Overall, most larvae completed development as expected within 2–10 days and settled permanently on the substrate, where they grew and completed their development as a primary polyp (Figure 7). However, some larvae underwent a metamorphosis in the water column (Figure 5), as previously observed by Richmond (1987), Mizrahi et al. (2014), and Barbosa, Vinagre, Mizrahi, and Flores (2019) for sun corals, and also for *P. damicornis* (Richmond, 1985).

This alternative life transition may occur as a response to the absence of a suitable substrate or to cues that inhibited normal settlement, such as the presence of cyanobacteria, sedimentation (Evensen, Doropoulos, Wong, & Mumby, 2019), and/or unfavorable water conditions (Ritson‐Williams et al., 2009). Although corals metamorphosed in the water column have not been observed in the field, in the aquaria they were able to feed, secrete a skeleton, and even start the benthic/sedentary phase when in contact with the substrate. Another remarkable sun coral reproductive strategy was the development of fused larvae that started benthic life already as a colony. Together, these alternative life cycles indicate wide developmental plasticity in *T. coccinea*, which probably plays a role in increasing its survival, spread, and population growth.

As a possible response to phenotypic plasticity and the amount of energy allocated to the offspring (Zera & Harshman, 2011), the dynamics of competence and the time spent in each developmental stage may vary widely across species and even within a given population (Davies, Meyer, Guermond, & Matz, 2014; Eckman, 1996). Larger larvae of *P. damicornis, S. hystrix,* and *S. pistillata* have longer life spans than smaller larvae, which may be advantageous for long‐distance dispersal (Isomura & Nishihira, 2001). The relative amount of energy investment in larvae may vary according to abiotic and biotic factors such as environmental stress and colony health, age, and size (Glynn et al., 2017; Hartmann, Marhaver, & Vermeij, 2018; Viladrich et al., 2017).

Despite the high larval longevity and competence, most *T. coccinea* offspring settle and undergo metamorphosis in a few days (~3–18 days; see also Harrison & Wallace, 1990; de Paula et al., 2014). However, the duration as motile larva varied by colony size (Figure 6) and was longer for the larvae from larger mother colonies, which suggests that colonies with 52 polyps or more may invest more energy in their offspring than the smaller colonies. This tendency may be a trade‐off between reproduction and survival and/or somatic growth of younger (smaller) colonies. Additionally, the larvae from smaller colonies usually displayed an aggregated settlement pattern near the parental colonies, as observed in several cases in Brazil (de Paula & Creed, 2005). Therefore, the release of different larval stages in addition to the rapid settlement capacity or longer period in the motile stage (in the water column) may represent a reproductive strategy that contributes to the invasiveness of *T. coccinea*, once it has settled into a new area.

Nevertheless, considering the variation in early developmental strategies of *T. coccinea*, the differences observed in the time needed to reach each stage as well as in the larval competence period may be the result of self‐fertilization, outcrossing, or asexual reproduction. If so, larvae originating from different reproductive modes may possess different amounts of energy reserves.

Independently of the reproductive strategy, early and mature larvae of *T. coccinea* were found simultaneously in the gastrovascular cavity; they were visible near the oral disk, close to the mouth, and less frequently inside the tentacles. Mature oocytes and embryos were observed at the base of the polyp. A similar distribution has been observed in other species of stony corals (e.g., *Cladopsammia willeyi* and *Astrangia danae*, by Szmant‐Froelich, Yevich, & Pilson, 1980) and soft corals (*A. glauca*, by Kruger et al., 1998; and *U. lindahli*, by Tyler et al., 1995). This distribution may allow more rapid expulsion of larvae by contraction of the oral disk, when environmental conditions occur that maximize larval survival and the chances of successful settlement.

## CONFLICT OF INTEREST

All authors state that there is no conflict of interest.

## AUTHOR CONTRIBUTIONS

**Bruna L. P. Luz:** Conceptualization (equal); data curation (lead); formal analysis (lead); investigation (lead); methodology (equal); writing – original draft (equal); writing – review & editing (equal). **Maikon Di Domenico:** Formal analysis (equal); methodology (equal); writing – original draft (equal); writing – review & editing (equal). **Alvaro E. Migotto:** Formal analysis (equal); investigation (equal); methodology (equal); resources (equal); writing – original draft (equal); writing – review & editing (equal). **Marcelo V. Kitahara:** Conceptualization (equal); funding acquisition (lead); investigation (equal); methodology (equal); project administration (lead); resources (equal); writing – original draft (equal); writing – review & editing (equal).

## ETHICAL APPROVAL

All applicable international, national, and/or institutional guidelines for the care and use of animals were followed.

## Data Availability

The dataset generated and analyzed during the current study is available in the Dryad repository (https://doi.org/10.5061/dryad.zw3r2285p).

## Footnotes

1 Tubastraea coccinea mature larvae released through a small pore at the tip of the tentacle. https://youtu.be/5dNVaD8ODNg

2 Tubastraea coccinea mature larvae released through a small pore at the tip of the tentacle. https://youtu.be/VA_rmMd‐Uww

3 Tubastraea coccinea mature larvae swimming freely inside the mother colony and more sporadically passing actively into and out of the mouth of the mother polyp. https://youtu.be/Rt0jBOIMeho

4 Tubastraea coccinea mature larvae expelled from the mother colony through “water jet”. https://youtu.be/6HBtBbPE618

5 Tubastraea coccinea mature larvae expelled from the mother colony through "induced" water jet. https://youtu.be/Yuauw966fNI

6 Tubastraea coccinea mature larvae expelled from the mother colony through gentle “water jet”. https://youtu.be/vhL‐uxrp7yk

7 Tubastraea coccinea larvae actively swimming out of the mother colony. https://youtu.be/jZmt2mRee7o

8 Active movement of the Tubastraea coccinea polyp's mesenteries exposing the planulae to the water column. https://youtu.be/cMGPeXCX3dw

9 Tubastraea coccinea released near formed larvae are motionless. https://youtu.be/87n2tSk‐ZJg

## References

[ece36346-bib-0001] Ayre, D. J. , & Hughes, T. P. (2000). Genotypic diversity and gene flow in brooding and spawning corals along the Great Barrier Reef, Australia. Evolution, 54, 1590–1605. 10.1111/j.0014-3820.2000.tb00704.x 11108587

[ece36346-bib-0002] Ayre, D. J. , & Resing, J. M. (1986). Sexual and asexual production of planulae in reef corals. Marine Biology, 90, 187–190. 10.1007/BF00569126

[ece36346-bib-0003] Baker, A. C. , Glynn, P. W. , & Riegl, B. (2008). Climate change and coral reef bleaching: An ecological assessment of long‐term impacts, recovery trends and future outlook. Estuarine, Coastal and Shelf Science, 80, 435–471. 10.1016/j.ecss.2008.09.003

[ece36346-bib-0004] Barbosa, A. C. C. , Vinagre, C. , Mizrahi, D. , & Flores, A. A. V. (2019). Temperature‐driven secondary competence windows may increase the dispersal potential of invasive sun corals. Marine Biology, 166, 131 10.1007/s00227-019-3580-7

[ece36346-bib-0005] Bartlett, M. S. (1937). Properties of sufficiency and statistical tests. Proceedings of the Royal Society of London, Series A: Mathematical and Physical Sciences, 160, 268–282. 10.1098/rspa.1937.0109

[ece36346-bib-0006] Bastidas, C. , Cróquer, A. , Zubillaga, A. L. , Ramos, R. , Kortnik, V. , Weinberger, C. , & Márquez, L. M. (2005). Coral mass‐ and split‐spawning at a coastal and an offshore Venezuelan reefs, southern Caribbean. Hydrobiologia, 541, 101–106. 10.1007/s10750-004-4672-y

[ece36346-bib-0007] Ben‐David‐Zaslow, R. , & Benayahu, Y. (1998). Competence and longevity in planulae of several soft corals. Marine Ecology Progress Series, 163, 235–243. 10.3354/meps171235

[ece36346-bib-0008] Benjamini, B. Y. , & Hochberg, Y. (1995). Controlling the false discovery rate: A practical and powerful approach to multiple testing. J R Statis Soc B, 57, 289–300.

[ece36346-bib-0009] Bishop, C. D. , Huggett, M. J. , Heyland, A. , Hodin, J. , & Brandhorst, B. P. (2006). Interspecific variation in metamorphic competence in marine invertebrates: The significance for comparative investigations into the timing of metamorphosis. Integrative and Comparative Biology, 46, 662–682. 10.1093/icb/icl043 21672777

[ece36346-bib-0010] Boschma, H. (1953). On specimens of the coral genus *Tubastraea*, with notes on phenomena of fission. Stud Fauna Curaçao Caribb Is, 29, 109–123.

[ece36346-bib-0011] Braendle, C. , Heyland, A. , & Flatt, T. (2011). Integrating mechanistic and evolutionary analysis of life history variation In Flatt T., & Heyland A. (Eds.), Mechanisms of life history evolution: The genetics and physiology of life traits and trade‐offs (1st ed., pp. 3–10). New York, NY: Oxford University Press.

[ece36346-bib-0012] Cairns, S. D. (1988). Asexual reproduction in solitary scleractinia, Australia. In: Proceedings of the 6th International Coral Reef Symposium (pp. 641–646).

[ece36346-bib-0013] Cairns, S. D. (1994). Scleractinia of the temperate North Pacific. Smithson Contrib Zool, 557, 1–150. 10.5479/si.00810282.557.i

[ece36346-bib-0014] Cairns, S. D. (2001). A generic revision and phylogenetic analysis of the Dendrophylliidae (Cnidaria: Scleractinia). Smithson Contrib Zool, 615, 1–75. 10.5479/si.00810282.615

[ece36346-bib-0015] Capel, K. C. C. , Creed, J. C. , & Kitahara, M. V. (2020). Invasive corals trigger seascape changes in the southwestern Atlantic. Bulletin of Marine Science, 96, 217–218. 10.5343/bms.2019.0075

[ece36346-bib-0016] Capel, K. C. C. , Creed, J. , Kitahara, M. V. , & Chen, C. A. , & Zilberberg, C. (2019). Multiple introductions and secondary dispersion of *Tubastraea* spp. in the Southwestern Atlantic. Scientific Reports, 9, 13978 10.1038/s41598-019-50442-3 31562380 PMC6765005

[ece36346-bib-0017] Capel, K. C. C. , Migotto, A. E. , Zilberberg, C. , & Kitahara, M. V. (2014). Another tool towards invasion? Polyp “bail‐out” in *Tubastraea coccinea* . Coral Reefs, 33, 1165 10.1007/s00338-014-1200-z

[ece36346-bib-0018] Capel, K. C. C. , Toonen, R. J. , Rachid, C. T. C. C. , Creed, J. C. , Kitahara, M. V. , Forsman, Z. , & Zilberberg, C. (2017). Clone wars: Asexual reproduction dominates in the invasive range of *Tubastraea* spp. (Anthozoa: Scleractinia) in the South‐Atlantic Ocean. PeerJ, 10, e3873 10.7717/peerj.3873 PMC5632532 29018611

[ece36346-bib-0019] Castro, C. B. , & Pires, D. O. (2001). Brazilian coral reefs: What we already know and what is still missing. Bulletin of Marine Science, 69, 357–371.

[ece36346-bib-0020] Conover, W. J. (1980). Practical nonparametric statistics (3rd ed.). New York, NY: Wiley.

[ece36346-bib-0021] Costa, T. J. F. , Pinheiro, H. T. , Teixeira, J. B. , Mazzei, E. F. , Bueno, L. , Hora, M. S. C. , … Rocha, L. A. (2014). Expansion of an invasive coral species over Abrolhos Bank, Southwestern Atlantic. Marine Pollution Bulletin, 85, 252–253. 10.1016/j.marpolbul.2014.06.002 24975092

[ece36346-bib-0022] Crabbe, M. J. C. (2008). Climate change, global warming and coral reefs: Modelling the effects of temperature. Computational Biology and Chemistry, 32, 311–314. 10.1016/j.compbiolchem.2008.04.001 18565794

[ece36346-bib-0023] Creed, J. C. (2006). Two invasive alien azooxanthellate corals, *Tubastraea coccinea* and *Tubastraea tagusensis*, dominate the native zooxanthellate *Mussismilia hispida* in Brazil. Coral Reefs, 25, 350 10.1007/s00338-006-0105-x

[ece36346-bib-0024] Creed, J. C. , Fenner, D. , Sammarco, P. , Cairns, S. D. , Capel, K. , Junqueira, A. O. R. R. , … Oigman‐Pszczol, S. (2017). The invasion of the azooxanthellate coral *Tubastraea* (Scleractinia: Dendrophylliidae) throughout the world: History, pathways and vectors. Biological Invasions, 19, 283–305. 10.1007/s10530-016-1279-y

[ece36346-bib-0025] Crowder, C. M. , Lo, L. W. , Weis, V. M. , & Fan, T. Y. (2014). Elevated temperature alters the lunar timing of planulation in the brooding coral *Pocillopora damicornis* . PLoS ONE, 9, e107906 10.1371/journal.pone.0107906 25329546 PMC4198079

[ece36346-bib-0026] Silva, A. G. , de Paula, A. F. , Fleury, B. G. , & Creed, J. C. (2014). Eleven years of range expansion of two invasive corals (*Tubastraea coccinea* and *Tubastraea tagusensis*) through the southwest Atlantic (Brazil). Estuarine, Coastal and Shelf Science, 141, 9–16. 10.1016/j.ecss.2014.01.013

[ece36346-bib-0027] Davies, S. W. , Meyer, E. , Guermond, S. M. , & Matz, M. V. (2014). A cross‐ocean comparison of responses to settlement cues in reef‐building corals. PeerJ, 2, e333 10.7717/peerj.333 24765568 PMC3994630

[ece36346-bib-0028] de Graaf, M. , Geertjes, G. J. , & Videler, J. J. (1999). Observations on spawning of scleractinian corals and other invertebrates on the reefs of Bonaire (Netherlands Antilles, Caribbean). Bulletin of Marine Science, 64, 189–194.

[ece36346-bib-0029] de Paula, A. F. , & Creed, J. C. (2004). Two species of the coral *Tubastraea* (Cnidaria, Scleractinia) in Brazil: A case of accidental introduction. Bulletin of Marine Science, 74, 175–183.

[ece36346-bib-0030] de Paula, A. F. , & Creed, J. C. (2005). Spatial distribution and abundance of nonindigenous coral genus *Tubastraea* (Cnidaria, Scleractinia) around Ilha Grande, Brazil. Brazilian Journal of Biology, 65, 661–673. 10.1590/S1519-69842005000400014 16532191

[ece36346-bib-0031] de Paula, A. F. , Fleury, B. G. , Lages, B. G. , & Creed, J. C. (2017). Experimental evaluation of the effects of management of invasive corals on native communities. Marine Ecology Progress Series, 572, 141–154. 10.3354/meps12131

[ece36346-bib-0032] de Paula, A. F. , Pires, D. O. , & Creed, J. C. (2014). Reproductive strategies of two invasive sun corals (*Tubastraea* spp.) in the southwestern Atlantic. Journal of the Marine Biological Association of the United Kingdom, 94, 481–492. 10.1017/S0025315413001446

[ece36346-bib-0033] Dunn, O. J. (1964). Multiple comparisons using rank sums. Technometrics, 6, 241–252. 10.1080/00401706.1964.10490181

[ece36346-bib-0034] Eckman, J. E. (1996). Closing the larval loop: Linking ecology to the population dynamics of marine benthic invertebrates. Journal of Experimental Marine Biology and Ecology, 200, 207–237.

[ece36346-bib-0035] Edmunds, P. J. , Cumbo, V. R. , & Fan, T.‐Y. (2013). Metabolic costs of larval settlement and metamorphosis in the coral *Seriatopora caliendrum* under ambient and elevated pCO_2_ . Journal of Experimental Marine Biology and Ecology, 443, 33–38. 10.1016/j.jembe.2013.02.032

[ece36346-bib-0036] Evensen, N. R. , Doropoulos, C. , Wong, K. J. , & Mumby, P. J. (2019). Stage‐specific effects of *Lobophora* on the recruitment success of a reef‐building coral. Coral Reefs, 38, 489–498. 10.1007/s00338-019-01804-w

[ece36346-bib-0037] Fadlallah, Y. H. (1983). Coral reefs sexual reproduction, development and larval biology in scleractinian corals. Coral Reefs, 2, 129–150. 10.1007/BF00336720

[ece36346-bib-0038] Fan, T. Y. , Li, J. J. , Ie, S. X. , & Fang, L. S. (2002). Lunar periodicity of larval release by *Pocilloporid* corals in southern Taiwan. Zoological Studies, 41, 288–293.

[ece36346-bib-0039] Fautin, D. G. (2002). Reproduction of Cnidaria. Canadian Journal of Zoology, 80, 1735–1754. 10.1139/z02-133

[ece36346-bib-0040] Fenner, D. (1999). New observations on the stony coral (Scleractinia, Milleporidae, and Stylasteridae) species of Belize (Central America) and Cozumel (Mexico). Bulletin of Marine Science, 64, 143–154.

[ece36346-bib-0041] Fenner, D. (2001). Biogeography of three Caribbean corals (Scleractinia) and the invasion of *Tubastraea coccinea* into the Gulf of Mexico. Bulletin of Marine Science, 69, 1175–1189.

[ece36346-bib-0042] Fenner, D. , & Banks, K. (2004). Orange cup coral *Tubastraea coccinea* invades Florida and the Flower Garden Banks, Northwestern Gulf of Mexico. Coral Reefs, 23, 505–507. 10.1007/s00338-004-0422-x

[ece36346-bib-0043] Gleason, D. F. , & Hofmann, D. K. (2011). Coral larvae: From gametes to recruits. Journal of Experimental Marine Biology and Ecology, 408, 42–57. 10.1016/j.jembe.2011.07.025

[ece36346-bib-0044] Glynn, P. W. , Colley, S. B. , Carpizo‐ituarte, E. , & Richmond, R. H. (2017). Coral reefs of the Eastern tropical Pacific In Glynn P. W., Manzello D. P., & Enochs I. C. (Eds.), Coral reefs of the eastern tropical Pacific, Coral reefs of the world (pp. 435–476). Dordrecht, The Netherlands: Springer Netherlands.

[ece36346-bib-0045] Glynn, P. W. , Colley, S. B. , Maté, J. L. , Cortés, J. , Guzman, H. M. , Bailey, R. L. , … Enochs, I. C. (2008). Reproductive ecology of the azooxanthellate coral *Tubastraea coccinea* in the Equatorial Eastern Pacific: Part V. Dendrophylliidae. Marine Biology, 153, 529–544. 10.1007/s00227-007-0827-5

[ece36346-bib-0046] Graham, E. M. , Baird, A. H. , & Connolly, S. R. (2008). Survival dynamics of scleractinian coral larvae and implications for dispersal. Coral Reefs, 27, 529–539. 10.1007/s00338-008-0361-z

[ece36346-bib-0047] Graham, E. M. , Baird, A. H. , Connolly, S. R. , Sewell, M. A. , & Willis, B. L. (2013). Rapid declines in metabolism explain extended coral larval longevity. Coral Reefs, 32, 539–549. 10.1007/s00338-012-0999-4

[ece36346-bib-0048] Graham, E. M. , Baird, A. H. , Connolly, S. R. , Sewell, M. A. , & Willis, B. L. (2017). Uncoupling temperature‐dependent mortality from lipid depletion for scleractinian coral larvae. Coral Reefs, 36, 97–104. 10.1007/s00338-016-1501-5

[ece36346-bib-0049] Graham, N. A. J. , & Nash, K. L. (2013). The importance of structural complexity in coral reef ecosystems. Coral Reefs, 32, 315–326. 10.1007/s00338-012-0984-y

[ece36346-bib-0050] Harrison, P. L. (2011). Sexual reproduction of scleractinian corals In Dubinsky Z., & Stambler N. (Eds.), Coral reefs: An ecosystem in transition (pp. 59–85). Dordrecht, The Netherlands: Springer.

[ece36346-bib-0051] Harrison, P. L. , Babcock, R. C. , Bull, G. D. , Oliver, J. K. , Wallace, C. C. , & Willis, B. L. (1984). Mass spawning in tropical reef corals. Science, 223, 1186–1189. 10.1126/science.223.4641.1186 17742935

[ece36346-bib-0052] Harrison, P. L. , & Wallace, C. C. (1990). Reproduction, dispersal and recruitment of scleractinian corals In Dubinsky Z. (Ed.), Coral reefs, ecosystems of the world (pp. 133–207). Amsterdam, The Netherlands: Elsevier.

[ece36346-bib-0053] Hartmann, A. C. , Marhaver, K. L. , & Vermeij, M. J. A. (2018). Corals in healthy populations produce more larvae per unit cover. Conservation Letters, 11, 1–12. 10.1111/conl.12410

[ece36346-bib-0054] Hayward, D. C. , Hetherington, S. , Behm, C. A. , Grasso, L. C. , Forêt, S. , Miller, D. J. , & Ball, E. E. (2011). Differential gene expression at coral settlement and metamorphosis – A subtractive hybridization study. PLoS ONE, 6, e26411 10.1371/journal.pone.0026411 22065994 PMC3204972

[ece36346-bib-0055] Heyland, A. , Degnan, S. , & Reitzel, A. M. (2011). Emerging patterns in the regulation and evolution of marine invertebrate settlement and metamorphosis In Flatt T., & Heyland A. (Eds.), Mechanisms of life history evolution: The genetics and physiology of life traits and trade‐offs (pp. 29–42). New York, NY: Oxford University Press.

[ece36346-bib-0056] Highsmith, R. C. (1982). Reproduction by fragmentation in corals. Marine Ecology Progress Series, 7, 207–226. 10.3354/meps007207

[ece36346-bib-0057] Hodin, J. (2006). Expanding networks: Signaling components in and a hypothesis for the evolution of metamorphosis. Integrative and Comparative Biology, 46, 719–742. 10.1093/icb/icl038 21672781

[ece36346-bib-0058] Hughes, K. A. , & Leips, J. (2017). Pleiotropy, constraint, and modularity in the evolution of life histories: Insights from genomic analyses. Annals of the New York Academy of Sciences, 1389, 76–91. 10.1111/nyas.13256 27936291 PMC5318229

[ece36346-bib-0059] Isaeva, V. V. , Akhmadieva, A. V. , Aleksandrova, Y. N. , Shukalyuk, A. I. , & Chernyshev, A. V. (2011). Germinal granules in interstitial cells of the colonial hydroids *Obelia longissima* Pallas, 1766 and Ectopleura crocea Agassiz, 1862. Russian Journal of Marine Biology, 37, 303–310. 10.1134/S1063074011040055

[ece36346-bib-0060] Isomura, N. , & Nishihira, M. (2001). Size variation of planulae and its effect on the lifetime of planulae in three pocilloporid corals. Coral Reefs, 20, 309–315. 10.1007/s003380100180

[ece36346-bib-0061] Kruger, A. , Schleyer, M. H. , & Benayahu, Y. (1998). Reproduction in *Anthelia glauca* (Octocorallia : Xeniidae). I. Gametogenesis and larval brooding. Marine Biodiversity Records, 131, 423–432.

[ece36346-bib-0062] Lages, B. G. , Fleury, B. G. , Menegola, C. , & Creed, J. C. (2011). Change in tropical rocky shore communities due to an alien coral invasion. Marine Ecology Progress Series, 438, 85–96. 10.3354/meps09290

[ece36346-bib-0063] Lin, K. (2005). Timing of larval release by five coral species in southern Taiwan: Seasonality, lunar and diurnal periodicity. Master's thesis, National Sun Yat‐Sen University, Taiwan.

[ece36346-bib-0064] Loya, Y. , & Rinkevich, B. (1980). Effects of oil pollution on coral reef communities. Marine Ecology Progress Series, 3, 167–180. 10.3354/meps003167

[ece36346-bib-0065] Luz, B. L. P. , Capel, K. C. C. , Zilberberg, C. , Flores, A. A. V. , Migotto, A. E. , & Kitahara, M. V. (2018). A polyp from nothing: The extreme regeneration capacity of the Atlantic invasive sun corals *Tubastraea coccinea* and *T. tagusensis* (Anthozoa, Scleractinia). Journal of Experimental Marine Biology and Ecology, 503, 60–65. 10.1016/j.jembe.2018.02.002

[ece36346-bib-0066] Luz, B. L. P. , & Kitahara, M. V. (2017). Could the invasive scleractinians *Tubastraea coccinea* and *T. tagusensis* replace the dominant zoantharian *Palythoa caribaeorum* in the Brazilian subtidal? Coral Reefs, 36, 875 10.1007/s00338-017-1578-5

[ece36346-bib-0067] McEdward, L. R. (2000). Adaptive evolution of larvae and life cycles. Seminars in Cell & Developmental Biology, 11, 403–409. 10.1006/scdb.2000.0193 11145868

[ece36346-bib-0068] Mizrahi, D. , Navarrete, S. A. , & Flores, A. A. V. (2014). Groups travel further: Pelagic metamorphosis and polyp clustering allow higher dispersal potential in sun coral propagules. Coral Reefs, 33, 443–448. 10.1007/s00338-014-1135-4

[ece36346-bib-0069] Nyström, M. , Folke, C. , & Moberg, F. (2000). Coral reef disturbance and resilience in a human‐dominated environment. Trends in Ecology & Evolution, 15, 413–417. 10.1016/S0169-5347(00)01948-0 10998519

[ece36346-bib-0070] Okubo, N. , Hayward, D. C. , Forêt, S. , & Ball, E. E. (2016). A comparative view of early development in the corals *Favia lizardensis*, *Ctenactis echinata*, and *Acropora millepora* – morphology, transcriptome, and developmental gene expression. BMC Evolutionary Biology, 16, 16–48. 10.1186/s12862-016-0615-2 26924819 PMC4770532

[ece36346-bib-0071] Okubo, N. , Mezaki, T. , Nozawa, Y. , Nakano, Y. , Lien, Y. T. , Fukami, H. , … Ball, E. E. (2013). Comparative embryology of eleven species of stony corals (Scleractinia). PLoS ONE, 8, e84115 10.1371/journal.pone.0084115 24367633 PMC3867500

[ece36346-bib-0072] Pineda, J. , Hare, J. , & Sponaugle, S. (2011). Larval transport and dispersal in the coastal ocean and consequences for population connectivity. Oceanography, 20, 22–39. 10.5670/oceanog.2007.27

[ece36346-bib-0073] Rapuano, H. , Brickner, I. , Shlesinger, T. , Meroz‐Fine, E. , Tamir, R. , & Loya, Y. (2017). Reproductive strategies of the coral *Turbinaria reniformis* in the northern Gulf of Aqaba (Red Sea). Scientific Reports, 7, 42670 10.1038/srep42670 28195203 PMC5307385

[ece36346-bib-0074] Richmond, R. (1985). Reversible metamorphosis in coral planula larvae. Marine Ecology Progress Series, 22, 181–185. 10.3354/meps022181

[ece36346-bib-0075] Richmond, R. H. (1987). Energetics, competency, and long‐distance dispersal of planula larvae of the coral *Pocillopora damicornis* . Marine Biology, 93, 527–533. 10.1007/BF00392790

[ece36346-bib-0076] Richmond, R. H. (1997). Reproduction and recruitment in corals: Critical links in the persistence of reefs In Birkeland C. (Ed.), Life and death of coral reefs (pp. 175–197). New York, NY: Springer.

[ece36346-bib-0077] Ritson‐Williams, R. , Arnold, S. N. , Fogarty, N. D. , Steneck, R. S. , Vermeij, M. J. A. , & Paul, V. J. (2009). New perspectives on ecological mechanisms affecting coral recruitment on reefs. Smithsonian Contributions to the Marine Sciences, 38, 437–457. 10.5479/si.01960768.38.437

[ece36346-bib-0078] Rodriguez, J. L. , Sedano, E. J. , García‐Martín, O. L. , Pérez‐Camacho, A. , & Sánchez, J. L. (1990). Energy metabolism of newly settled *Ostrea edulis* spat during metamorphosis. Marine Biology, 106, 109–111. 10.1007/BF02114680

[ece36346-bib-0079] Romano, S. L. , & Cairns, S. D. (2000). Molecular phylogenetic hypotheses for the evolution of scleractinian corals. Bulletin of Marine Science, 67, 1043–1068.

[ece36346-bib-0080] Royston, J. P. (2006). An extension of Shapiro and Wilk's W test for normality to large samples. Applied Statistics, 31, 115 10.2307/2347973

[ece36346-bib-0081] Sammarco, P. W. (1982). Polyp bail‐out: An escape response to environmental stress and a new means of reproduction in corals. Marine Ecology Progress Series, 10, 57–65. 10.3354/meps010057

[ece36346-bib-0082] Sammarco, P. W. , Porter, S. A. , & Cairns, S. D. (2010). A new coral species introduced into the Atlantic Ocean ‐*Tubastraea micranthus* (Ehrenberg 1834) (Cnidaria, Anthozoa, Scleractinia): An invasive threat? Aquatic Invasions, 5, 131–140. 10.3391/ai.2010.5.2.02

[ece36346-bib-0083] Schnitzler, C. E. , Hollingsworth, L. L. , Krupp, D. A. , & Weis, V. M. (2012). Elevated temperature impairs onset of symbiosis and reduces survivorship in larvae of the Hawaiian coral, *Fungia scutaria* . Marine Biology, 159, 633–642. 10.1007/s00227-011-1842-0

[ece36346-bib-0084] Sewell, M. A. (2005). Utilization of lipids during early development of the sea urchin *Evechinus chloroticus* . Marine Ecology Progress Series, 304, 133–142. 10.3354/meps304133

[ece36346-bib-0085] Shanks, A. L. (2009). Pelagic larval duration and dispersal distance. Biological Bulletin, 216, 373–385. 10.2307/25548167 19556601

[ece36346-bib-0086] Sherman, C. D. H. , Ayre, D. J. , & Miller, K. J. (2006). Asexual reproduction does not produce clonal populations of the brooding coral *Pocillopora damicornis* on the Great Barrier Reef, Australia. Coral Reefs, 25, 7–18. 10.1007/s00338-005-0053-x

[ece36346-bib-0087] Shikina, S. H. , & Chang, C. F. (2016). Sexual reproduction in stony corals and insight into the evolution of oogenesis in Cnidaria In Goffredo S., & Dubinsky Z. (Eds.), The Cnidaria, past, present and future: The world of Medusa and her sisters (pp. 249–268). Basel, Switzerland: Springer Nature.

[ece36346-bib-0107] Silva, A. G. Da , Paula, A. F. De , & Creed, J. C. (2014). Eleven years of range expansion of two invasive corals (Tubastraea coccinea and Tubastraea tagusensis) through the southwest Atlantic (Brazil). Estuar Coast Shelf Sci, 141(9), 16 10.1016/j.ecss.2014.01.013

[ece36346-bib-0088] Silva, R. , Vinagre, C. , Kitahara, M. V. , Acorsi, I. V. , Mizrahi, D. , & Flores, A. A. V. (2019). Sun coral invasion of shallow rocky reefs: Effects on mobile invertebrate assemblages in Southeastern Brazil. Biological Invasions, 21, 1339–1350. 10.1007/s10530-018-1903-0

[ece36346-bib-0089] Sorek, M. , & Levy, O. (2014). Coral spawning behavior and timing In Numata H., & Helm B. (Eds.), Annual, lunar, and tidal clocks: Patterns and mechanisms of nature's enigmatic rhythms (pp. 81–98). Tokyo, Japan: Springer.

[ece36346-bib-0090] Stearns, S. C. (1992). The evolution of life histories (12th ed.). New York, NY: Oxford University Press.

[ece36346-bib-0091] Stearns, S. C. (2000). Life history evolution: Successes, limitations, and prospects. Naturwissenschaften, 87, 476–486. 10.1007/s001140050763 11151666

[ece36346-bib-0092] Steiner, S. C. C. (1995). Spawning in Scleractinian corals from SW Puerto Rico (West Indies). Bulletin of Marine Science, 56, 899–902.

[ece36346-bib-0093] Strader, M. E. , Aglyamova, G. V. , & Matz, M. V. (2018). Molecular characterization of larval development from fertilization to metamorphosis in a reef‐building coral. BMC Genomics, 19, 17 10.1186/s12864-017-4392-0 29301490 PMC5755313

[ece36346-bib-0094] Strathmann, R. R. (1986). What controls the type of larval development? Summary statement for the evolution session. Bulletin of Marine Science, 39, 616–622.

[ece36346-bib-0095] Szmant‐Froelich, A. , Yevich, P. , & Pilson, M. E. Q. (1980). Gametogenesis and early development of the temperate coral *Astrangia danae* (Anthozoa: Scleractinia). Biological Bulletin, 158, 257–269.

[ece36346-bib-0096] Tyler, P. A. , Bronsdon, S. K. , Young, C. M. , & Rice, A. L. (1995). Ecology and gametogenic biology of the genus *Umbellula* (Pennatulacea) in the North Atlantic Ocean. Internationale Revue Der Gesamten Hydrobiologie Und Hydrographie, 80, 187–199. 10.1002/iroh.19950800207

[ece36346-bib-0097] Vaughan, T. W. , & Wells, J. W. (1943). Revision of the suborders, families and genera of the Scleractinia. Special Papers of the Geological Society of America, 44, 1–363. 10.1130/SPE44-p1

[ece36346-bib-0098] Vermeij, M. J. A. , Barott, K. L. , Johnson, A. E. , & Marhaver, K. L. (2010). Release of eggs from tentacles in a Caribbean coral. Coral Reefs, 29, 411 10.1007/s00338-010-0595-4

[ece36346-bib-0099] Vermeij, M. J. A. , Sampayo, E. , Bröker, K. , & Bak, R. P. M. (2004). The reproductive biology of closely related coral species: Gametogenesis in *Madracis* from the southern Caribbean. Coral Reefs, 23, 206–214. 10.1007/s00338-004-0368-z

[ece36346-bib-0100] Viladrich, N. , Bramanti, L. , Tsounis, G. , Martínez‐Quitana, A. , Ferrier‐Pagès, C. , & Rossi, S. (2017). Variation of lipid and free fatty acid contents during larval release in two temperate octocorals according to their trophic strategy. Marine Ecology Progress Series, 573, 117–128. 10.3354/meps12141

[ece36346-bib-0101] Ward, S. (1992). Evidence for broadcast spawning as well as brooding in the scleractinian coral *Pocillopora damicornis* . Marine Biology, 112, 641–646. 10.1007/BF00346182

[ece36346-bib-0102] Wendt, D. E. (2000). Energetics of larval swimming and metamorphosis in four species of *Bugula* (Bryozoa). Biological Bulletin, 198, 346–356. 10.2307/1542690 10897448

[ece36346-bib-0103] Whalan, S. , Johnson, M. S. , Harvey, E. , & Battershill, C. (2005). Mode of reproduction, recruitment, and genetic subdivision in the brooding sponge *Haliclona* sp. Marine Biology, 146, 425–433. 10.1007/s00227-004-1466-8

[ece36346-bib-0104] Willis, B. L. , & Oliver, J. K. (1990). Direct tracking of coral larvae: Implications for dispersal studies of planktonic larvae in topographically complex environments. Ophelia, 32, 145–162. 10.1080/00785236.1990.10422029

[ece36346-bib-0105] Wood, S. , Baums, I. B. , Paris, C. B. , Ridgwell, A. , Kessler, W. S. , & Hendy, E. J. (2016). El Niño and coral larval dispersal across the eastern Pacific marine barrier. Nature Communications, 7, 12571 10.1038/ncomms12571 PMC4996977 27550393

[ece36346-bib-0106] Zera, A. J. , & Harshman, L. G. (2011). Intermediary metabolism and the biochemical‐molecular basis of life history variation and trade‐offs in two insect models In Flatt T., & Heyward A. J. (Eds.), Mechanisms of life history evolution: The genetics and physiology of life traits and trade‐offs (pp. 311–328). New York, NY: Oxford University Press.

